# Quality of Informed Consent in Mammography Screening—The Polish Experience

**DOI:** 10.3390/ijerph19116735

**Published:** 2022-05-31

**Authors:** Anna Zagaja, Renata Bogusz, Jarosław Sak, Michał Wiechetek, Jakub Pawlikowski

**Affiliations:** 1 Chair and Department of Humanities and Social Medicine, Medical University of Lublin, 20-059 Lublin, Poland; renata.bogusz@umlub.pl (R.B.); jareksak@o2.pl (J.S.); jpawlikowski@wp.pl (J.P.); 2 Institute of Psychology, The John Paul II Catholic University of Lublin, 20-950 Lublin, Poland; michal.wiechetek@kul.pl

**Keywords:** quality of informed consent, benefit–risk ratio, mammography, breast cancer screening

## Abstract

Breast cancer is one of the leading forms of cancers in women worldwide. In Poland, it accounts for approx. 20% of all cancers diagnosed, with approximately 11,000 new cases and 5000 deaths from this disease annually. To prevent unfavourable statistics, Poland introduced free breast cancer screening programmes, available to women aged 50–69. Over a million women take advantage of this programme each year. The aim of the research was to assess the quality of consent women give prior to mammography screening and address the question of whether this quality is sufficient to make an informed choice. The study was conducted on a representative group of 600 Polish women over 50 years old (475 of them had undergone mammography screening), who agreed to take part in the study. Using the computer-assisted interview technology (CATI) method, all women were asked about their perception of breast cancer and screening and those who had undergone mammography were quizzed about the consent process. They will form the focus of this research. The validated tool contained items on both the benefits and risks of screening. The results indicate that the quality of informed consent was insufficient. A discrepancy was observed in the awareness between the benefits and risks of mammography screening. The main motivations to undergo screening were: prophylactic purposes and the free-of-charge nature of this health service. Population-based screening programmes for breast cancer should be reconsidered in terms of information policy, and the quality of informed consent should be increased.

## 1. Introduction

According to data from the International Agency for Research on Cancer (IARC) of the World Health Organization (WHO), breast cancer affects about 2.1 million women worldwide annually. Despite steady mortality rates, an increase in incidence is observed, which is also apparent in Poland [1]. The highest increase in the risk of developing the disease is observed among women aged 50–69. Statistically, as much as 80% of cases occur after the age of 50 and almost 50% are diagnosed between the ages of 50 and 69. To enable early treatment, emphasis is placed on prevention and early diagnosis. At the turn of the 1980s and 1990s, individual countries had already begun to introduce preventive programmes aimed at the early detection of breast cancer [2]. In Poland, this prophylactic approach encompasses women aged 50–69 and mammography is recommended once every two years (unless individually recommended otherwise) [3]. As of 1 February 2020, the average mammography population coverage had reached 37.15% (by 1 February 2021, it had decreased by 4.63 percentage points) [4]. Mammography has been considered a beneficial measure for reducing breast cancer mortality through the detection of the preclinical stages of the disease. What is more, it allows for the monitoring of incidence and assures women of their health status. Despite technical advances over the past few decades, however, there are still limitations to the mammography technique and screening programmes. These apply to, inter alia, false-positive and false-negative results, overdiagnosis and psychological as well as physical and economic burden. In light of the above concerns, it is extremely important that women are provided with risk information essential to evaluating their own state of health and to making autonomous informed decisions.

For decades, medical ethics and medical law have emphasised the importance of respecting patients’ autonomy, and one of the basic manifestations of this respect is the need to obtain informed consent for health services. Recently, a discussion not only on whether consent should be granted but also on the quality of such consent has arisen. Quality of consent is understood as the compilation of all factors influencing the process of granting consent. This includes the scope and method of providing information, as well as ensuring appropriate conditions in terms of place, time and circumstances. Providing balanced information (including an assessment of the benefit–risk ratio), making sure that the patient understood the provided data and receiving a signature (if required) on the consent form may be tantamount to obtaining high-quality consent. It would seem that these theoretical assumptions will also work in practice. Nevertheless, high-quality consent remains an ongoing challenge in various areas of medicine, including clinical trials [5], surgical procedures [6], vaccination [7] and blood transfusion [8]. In each of these areas of medicine, the process of obtaining consent has been determined and it is a gateway to further, frequently necessary, treatment.

In the case of screening tests, neither formal nor content requirements for obtaining consent are specified. Neither the process of obtaining nor the scope of the information provided is regulated, and receiving consent from a person wishing to undergo screening is not standard. Paradoxically, screening is one of the few areas of medicine where high-quality informed consent could be easily obtained, because it concerns healthy people, or at least asymptomatic ones, who chose to learn about their current state of health. In research literature, concerns have been raised as to the quality of leaflets and brochures for mammography screening, underlining their data selection bias and the problem of readability [9,10]; however, the quality of consent for breast cancer screening still requires in-depth analysis.

The aim of the research was to determine the quality of consent expressed by Polish women prior to undergoing mammography screening. This is important for improving conscious participation and public trust in screening. The study was based on an approach developed by K.J. Jørgensen and P.C. Gøtzsche in 2006 for assessing the quality of expressed consent for mammography screening [9]. Initially, the criteria concerned leaflets and information materials, but the authors indicated that it was insufficient to assess women’s quality of consent due to the information that women can obtain immediately prior to the screening [9]. In our study, we used modified criteria to check the quality of consent of women who underwent screening in order to eliminate this uncertainty.

## 2. Materials and Methods

The survey was conducted using the computer-assisted telephone interview method in a nationwide survey of a representative group of 600 women aged 50+ by an external, qualified, survey company. The distribution of respondents was developed based on the criteria of age (three age groups: 50–60, 60–70 and over 70) and place of residence (three groups: city with over 100 thousand inhabitants, city with under 100 thousand inhabitants and rural areas) based on generally available statistics from the Central Statistical Office. The database of respondents’ numbers was a set of mobile and landline phone numbers, randomly generated by the CATI survey system, in a ratio 80:20. The tele-interviewer first presented the purpose of the interview and formulated a request for consent to participate in the study. Lack of consent ended contact with the respondent, while securing consent triggered the procedure of verifying the respondent in terms of the assumed sociodemographic characteristics. Positive verification was a precondition for examination. The response rate for the study was 38%. There was also a 2% interruption rate for interviews that had already begun. The maximum adopted statistical error was 4% with a 95% confidence interval.

The inclusion criteria were sex (women), age (50 and above) and previous mammography experience. The exclusion criteria were sex (men), age (women below 50), women who lacked mammography experience and those that did not agree to take part in the study. Overall, 600 women agreed to take part in the study, 475 of whom had undergone mammography screening in the past (the majority had undergone the screening over a year ego (44%), 22% within the preceding 6 months, 15% within the preceding year, and 19% within the last month). Most of the respondents were in the 50–60 age group. Most (79%) came from cities and had secondary (42%) and vocational (40%) education. Four percent of the respondents had medical education. About a third (32%) had a breast cancer history in their family. A significant percentage (54%) declared that they had a suspicion of breast cancer for themselves, and 33% had been diagnosed with the disease at some point in their lifetime.

The study was divided in two parts: women’s perception of breast cancer (total group) and quality of consent (only women who took part in screening). In this study, we present only the results regarding the quality of consent. The survey used a questionnaire containing a validated tool to assess the quality of consent to mammography screening preceded by sociodemographic questions. The tool for assessing the quality of expressed consent was based on the research conducted by K.J. Jørgensen and P.C. Gøtzsche in 2006, who developed the criteria regarding the scope of information necessary to obtain informed consent for mammography screening [9]. Two types of criteria were taken into account for assessing the quality of informed consent: formal criteria (regarding the circumstances of receiving consent, the opportunity to ask questions, information about the risks and benefits, written form, feedback, etc.) and content criteria (types of benefits and risks, the scope of information, etc.). Additionally, an open question on the motives for undergoing screening was asked. The authors analysed three screening methods offered in Poland: public ambulatories, private practices and mammobuses.

The results were processed using both SPSS and the Statistica statistical package. Descriptive statistics, such as mean (M) and standard deviation (SD), were used, and the frequencies of different categories of consent (N) and percentage (%) were calculated. One-way analysis of variance along with Tukey’s post hoc tests and effect size (eta) assessment were used to test for differences in the global level of consent quality.

The research process was initiated upon obtaining the approval of the Bioethics Committee at the Medical University of Lublin (KE-0254/228/2016).

## 3. Results

The research results indicate that some criteria for the high quality of informed consent were met to a large extent, while others were not. In terms of the formal criteria, the majority of women declared that consent was received in comfortable conditions (72%), they were asked to document their consent in writing (62%), they were informed about the benefits (71%) or they had the opportunity to ask questions prior to giving consent (52%). A minority of the women were informed about the risks associated with mammography (35%) or declared that the person who obtained consent had not asked for their feedback regarding whether they had understood the information supplied before providing a written signature on the consent form (Table 1).

Assessment of the content criteria revealed that participants were well informed about benefits but many had not had enough information to label the consent given as sufficiently “informed.” Only one in four women knew about the risks of obtaining ambiguous results, and about one-third were aware of the possibility of obtaining a false-positive (31%) and a false-negative result (28%). Less than half (43%) of the respondents knew about the possibility of interval cancer, and an additional 10% knew about the possibility of detecting a non-progressive disease (overdiagnosis) (Table 2). What was interesting was that women who had booked their screening in mammobuses were better informed.

During the analysis, the authors also determined which variables modify the global quality of informed consent in the study group. For this purpose, a global index of the quality of consent was calculated, assigning 1 point to each answer indicating the fulfilment of formal and content criteria of consent expressed prior to mammographic screening. If these conditions were not fulfilled or if the respondent stated that she does not recall the fulfilment of these criteria, 0 points were assigned. The total value of this index in the study group ranged from 2 to 13 points (M = 6.92; SD = 1.96). The study revealed differences in the level of the quality of informed consent on account of two sociodemographic variables: age and place of residence (Table 3). In the case of age, the highest quality of consent was obtained from respondents from the 50–60 years group and the lowest from respondents over 70. The quality of consent from respondents between 61 and 70 was not statistically significantly different from either that from the younger or that from the older group of respondents. Statistically significant differences were visible in terms of the place of residence. Here, the highest level of informed consent was obtained from women living in rural areas. The lowest was from those from cities with more than 100,000 inhabitants. Statistical differences were also observed in association with inhabitants from rural areas, as well as cities with fewer than 10,000 inhabitants.

**Figure 1:**
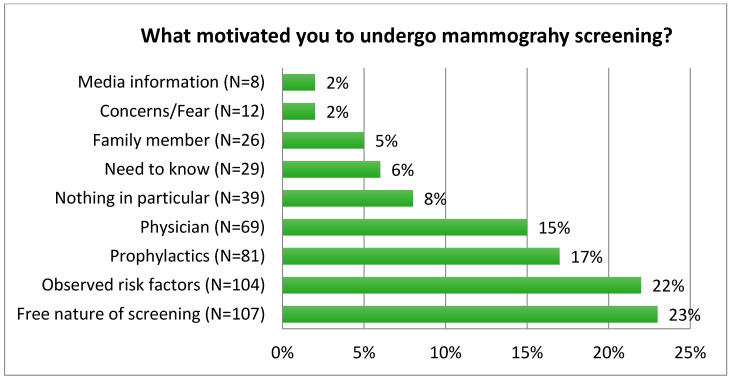
Motives behind undergoing mammography screening.

Interesting results were obtained in the open question regarding the motives behind undergoing mammography. Each woman gave the most important, in her opinion, reason for attending screening. Those reasons were grouped into nine categories. The most frequently mentioned premise was the free nature of the mammography screening, while observed risk factors, including breast pain, lumps or age came second. Some women (15%) stated that they had been advised by their doctor. The smallest number of respondents (2%) indicated information on the media, as well as general concerns/fears (Figure 1).

## 4. Discussion

The increase in the incidence of breast cancer is a challenge for modern medicine and public health; therefore, measures should be taken to improve prevention, early detection, treatment and rehabilitation. Apart from promoting a healthy lifestyle, providing health education, building awareness around the risk factors and shaping the habit of self-examination, a common method of early detection of breast cancer is screening, organised either according to the population model or the opportunistic model. In Poland, the population model has been adopted, in which all women aged 50 to 69 are offered free mammographic exams. Currently, the benefits of population screening are still being questioned; therefore, it is extremely important that women make decisions about screening on the basis of reliable information and not social stereotypes advocating or negating the value of screening. Although research by Bogusz et al. indicates that respondents expect information on mammography to be propagated by medical staff, mainly by doctors (77%) (e.g., general practitioners and gynaecologists) [11], encouragement by a doctor was provided as a motivating factor only by 15% of the respondents. This may suggest that the physician does not play such an important role in motivating screenings or that medical personnel are not such an important source of information but it is the free nature of screening that is a key motivator.

In this research, as many as 65% of respondents received information leaflets; however, taking into account previous reports and the content of information contained in available promotional materials, it is difficult to assess whether those leaflets were a reliable source of information, sufficient to make an informed decision. Analyses of brochures from different countries have revealed a number of instances of ambiguous or even misleading information. It has been noticed that the information on benefits is maximised, while the information on the risks of mammography screening is minimised [10]. A similar problem was identified in mammography invitations. Research by Jørgensen and Gøtzsche revealed that invitations from selected countries included on average only 2 to 6 out of the 16 pieces of information necessary for informed consent [9]. The results of research carried out by Gummersbach [10], Gøtzsche [9], Zapka [12] and Ballesteros-Peña [13] indicate a large similarity in information shortage across the analysed leaflets/brochures, regardless of differences resulting from the medical culture of a given country. Although the information contained in these leaflets and forms, and the research was conducted in the past, the problem of information shortage, as demonstrated by this study, is still valid. What is more, since 2015, Polish women no longer receive invitations to attend screenings; therefore, control of the information provided is limited and the route to obtain it is cut short. Nowadays, the number of publications on the phenomenon of overdiagnosis, false-negative results, interval cancers and the stress of obtaining false-positive results is increasing [14,15,16]. Studies have shown that in North America, the false-positive rates associated with screening programmes vary from 10.2% to 14.4% [17]. Moreover, studies based on a retrospective analysis indicate that many of the missed cancers can be found in previous mammograms [18]. Such errors in mammographic assessments may result in legal sanctions; therefore, radiologists may seek to identify each imaged lesion. This, in turn, may expose women to additional diagnostic/therapeutic interventions and reduce their quality of life [19]. In addition to the mental and health burden, the financial costs associated with obtaining a false-positive result should also not be underestimated [20]. It might seem that with the passage of time and an increasing amount of information on false-positive results, women would be made more aware of this phenomenon. This research has shown, however, that awareness of false-positive results among women who have had a mammogram is only 30%. This, in turn, may suggest that most women who undergo mammography believe that a positive test result is synonymous with a cancer diagnosis. Further, articles raising concerns as to mammography-induced cancer have begun to appear [21,22,23,24]. What is more, cancer diagnosis at a preclinical stage of the disease may lead to overdiagnosis. Given that current screening technologies are aimed at detecting preclinical changes, it can be assumed that some level of overdiagnosis in breast cancer screening will be the rule rather than an exception [25,26]. The available data indicate that the level of overdiagnosis varies, and according to some it may be as high as 50% [27,28,29,30,31,32,33]. Overdiagnosis is the phenomenon where a diagnosis is made suggesting a more serious clinical condition than the actual one [34]. For the patient, this means introducing disproportionate treatment (overtreatment) and living with the awareness of a disease, which in turn may lead to unnecessary mental stress (anxiety, depression, etc.), physical effects induced by medical interventions (e.g., chemotherapy, radiotherapy or oncological treatments) and financial burden, as well as to unjustified expenses for the state.

Analysis of the epidemiological situation shows that in Poland (compared to other European countries) there is higher mortality with lower incidence rates. This may be down to two reasons: (1) improperly conducted prevention programmes [35] and (2) low reporting rates. The overdiagnosis rate is not as high as in countries where attendance for mammography screening is higher. Regardless of the lack of consensus as to the reasons for obtaining such results, information about the possibility of non-progressive changes should be made available to all women who decide to undergo screening, even if it may result in a reluctance to screen [34]. This study revealed a large disproportion between the perception of the risks and benefits of mammography screening. Women tend to have a much higher level of knowledge about the benefits compared to the risks and these results seem to be confirmed, e.g., in research conducted by Yu, where most of the respondents knew the benefits of mammography and assessed them as important, while their knowledge and assessment of potential risks varied and was at a lower level [36]. Moreover, knowledge influences attitudes and decisions—women who see more benefits are more likely to take advantage of screening, perform it regularly and accept more drawbacks (e.g., discomfort). Undoubtedly, information about the risks could discourage some women from participating in the research and reduce the scope of the population covered. A conflict arises here between respecting a patient’s autonomy and the effectiveness of preventive programmes. Nevertheless, a situation in which women are not aware of risks, when it would be feasible to be informed fully, bears the hallmarks of paternalism, is a manifestation of lack of respect for autonomy and is a denial of the standards and values underlying informed consent. The results of this research indicate shortcomings in both formal and content requirements. Therefore, as essential elements are omitted, it is impossible to argue that the level of quality of informed consent is high. Although obtaining consent in writing is not a legal requirement for mammography screening, there is no doubt that the ethical standard of conduct prescribes consent (expressed orally or in writing) which is based on reliable information and balanced content regarding both benefits and risks. In the modern paradigm of medical ethics and medical law, respect for the principle of autonomy is a fundamental assumption. Therefore, informed decision-making should be standard not only in clinical practice but also in public health activities. A realistic perception of mammography screening and concern for high-quality consent may paradoxically contribute to an increase in the effectiveness of screening for breast cancer. Screening is most beneficial when it detects the most potentially dangerous lesions that can be effectively treated at this stage. Therefore, perhaps, women who are better informed about cancer and screening will make more informed decisions about prevention by analysing their individual health situation rather than succumbing to peer pressure, catchy advertisements or motivation related to free testing. It is also hoped that the standards of informed consent that have been used for years in clinical trials and other medical experiments, as well as in clinical practice, will also cover preventive interventions in public health.

## 5. Limitations

The study is not devoid of limitations. As it was not an on-site study, it is difficult to differentiate between what women were told prior to screening during consent acquisition and what they already knew. If respondents relied on knowledge obtained from different sources, this would indicate that the quality of obtained consent was even lower.

## 6. Conclusions

The conducted research as well as the analysis and discussion of the results allow the following conclusions to be drawn:

1. Consent for mammography in many cases did not meet the content (including information on overdiagnosis and false-positive results) and formal criteria, which reduced its quality. Effort should be made to raise the standard of obtaining consent for mammography screening.
2. The main motivations for women to undergo mammography screening were to reduce their concerns about observed risk factors and to avail of the free screening.
3. Effort should be made to balance the benefit–risk ratio. Overestimation of benefits and underestimation of risks in mammography perception can lead to unrealistic expectations and emotional overburden. As the process of obtaining consent is difficult and time consuming, it would be best to combine some elements of it, e.g., by sending patients balanced information along with the screening invitation.

## Figures and Tables

**Table 1:** Formal criteria of consent.

	Yes (%)	No (%)	I Do Not Remember (%)
Consent was obtained in comfortable conditions (peace, quiet).	72.4	10.3	17.3
Prior to screening, I was asked to sign a consent form.	61.9	19.4	18.7
I received information materials on screening (leaflets, brochures, etc.).	64.9	16.6	18.5
Prior to providing my consent, I was informed orally about the benefits associated with mammography.	70.7	7	22.3
Prior to providing my consent, I was informed orally about the risks associated with mammography.	34.9	41.9	23.2
The person obtaining consent made sure that I understood the information provided (asked questions, feedback, etc.) before obtaining my signature on the consent form.	29.7	29.3	41
I had the opportunity to ask questions prior to undergoing screening.	52.4	17.5	30.1

**Table 2:** Content criteria of consent.

	Yes		No		I Do Not Remember	
	N	%	N	%	N	%
Before screening, I was informed of its benefits e.g., greater effectiveness of treatment if cancer is detected at an earlier stage.	292	61.5	56	11.8	127	26.7
Before mammography, I was informed of the risk of ambiguous results.	119	25	144	30.3	212	44.7
Before mammography, I was informed of the risk of an erroneous result suggesting the presence of breast cancer despite its absence.	145	30.5	144	30.5	186	39
Before mammography, I was informed of the risk of an erroneous result suggesting no breast cancer despite its actual presence (or the risk of missing a cancer).	132	27.8	169	35.6	174	36.6
Before mammography, I was informed of the possibility of my developing cancer between screening tests.	207	43.6	132	27.8	136	28.6
Before mammography, I was informed of the possibility of the detection of minor changes that carry no risk of death (overdiagnosis).	252	53	94	19.8	129	27.2
Before mammography, I was informed of the recommendation for self-examination.	304	64	31	6.5	140	29.5

**Table 3:** Sociodemographic factors modifying activities aimed at obtaining informed consent—results of one-way analysis of variance.

Variable	M	SD	F	p	Post Hoc	Effect Size
Age						
50–60 (1)	7.05	1.89	3.215	0.041	1 > 3	0.013
61–70 (2)	7.01	1.98				
70+ (3)	6.46	2.08				
Place of residence						
Countryside (1)	7.19	1.96	5.655	0.004	1 > 3; 2 > 3	0.023
City with up to 100 thousand (2)	7.18	1.90				
City with over 100 thousand (3)	6.58	1.97				
Education						
Primary education (1)	7.16	1.80	1.330	0.264	–	0.008
Vocational (2)	7.09	1.99				
High school (3)	6.88	1.96				
Higher (4)	6.57	1.93				

## Data Availability

Data available on reasonable request.
